# Ictal gamma increase shows preferential localization to seizure onset contacts in stereoelectroencephalography

**DOI:** 10.3389/fneur.2026.1862336

**Published:** 2026-05-29

**Authors:** Yunbo Jian, Kaifeng Hou, Wenqian Zhang, Lin Wang, Jianye Wang

**Affiliations:** 1 Department of Neurosurgery, Xuanwu Hospital, Capital Medical University, Beijing, China; 2 Department of Neurosurgery, The Fifth Affiliated Hospital, Zhengzhou University, Zhengzhou, China; 3 Capital Institute of Pediatrics, Beijing, China; 4 Department of Neurology, The Fifth Affiliated Hospital, Zhengzhou University, Zhengzhou, China

**Keywords:** epilepsy, gamma oscillation, ictal dynamics, localization, seizure onset zone, stereoelectroencephalography

## Abstract

**Objective:**

To quantify ictal gamma dynamics in a large stereoelectroencephalography (SEEG) dataset and determine whether ictal gamma increase is preferentially associated with seizure onset zone (SOZ) contacts.

**Methods:**

Seizures from the public OpenNeuro dataset ds004100 were analyzed. Gamma-band activity (30–80 Hz) was quantified for each contact as the log2-transformed ratio of ictal gamma power to 5-min preictal baseline power. Gamma dynamics were summarized at the contact, seizure, and patient levels. SOZ-related differences were evaluated using pooled descriptive comparisons, within-patient analyses, and hierarchical mixed-effects modeling. We also tested whether the top 10% gamma contacts within each seizure were enriched in SOZ contacts and performed coordinate-informed region-stratified analyses.

**Results:**

A total of 16,262 contacts, 138 seizures, and 36 patients were included. SOZ contacts showed higher gamma log-ratio values than non-SOZ contacts (median 1.838 vs. 0.696, *p* = 9.46 × 10^-23). In hierarchical mixed-effects modeling, SOZ status remained significantly associated with higher contact-level gamma log-ratio values (*β* = 0.802, 95% CI 0.739 to 0.864, *p* < 2 × 10^-16). Top-gamma contacts were enriched in SOZ contacts (28.8% vs. 6.7%, odds ratio 5.65, *p* = 2.92 × 10^-123). Region-stratified analyses showed the strongest SOZ versus non-SOZ separation in mesial temporal contacts, with additional significant differences in lateral temporal, insular, parietal, and central sensorimotor regions.

**Conclusion:**

Ictal gamma activity increases during seizures and is consistently associated with seizure onset contacts, with anatomically non-uniform SOZ-related effects across sampled regions.

**Significance:**

These findings suggest that ictal gamma increase may provide spatially informative electrophysiological information related to seizure onset contacts in intracranial recordings.

## 1 Introduction

Accurate localization of seizure onset-related activity remains central to the interpretation of intracranial EEG, particularly stereoelectroencephalography (SEEG), and to the presurgical evaluation of drug-resistant epilepsy (1). Although clinical interpretation relies heavily on visual assessment of seizure onset patterns, quantitative electrophysiological markers may provide complementary information about seizure recruitment and spatial organization (2, 3). Among these, gamma-band activity has attracted sustained interest because it may reflect local neuronal population recruitment and evolving ictal synchronization (4, 5).

Prior studies have reported increased gamma activity during seizures and suggested possible links with seizure onset regions, ictal propagation, and epileptogenic networks (6, 7). However, the magnitude and spatial distribution of ictal gamma increase appear to vary substantially across seizures and across patients (8, 9). In many studies, sample sizes have been modest, analyses have been limited to a single scale of observation, or localization-related comparisons have not been systematically evaluated across large numbers of contacts (10, 11).

The increasing availability of open intracranial EEG datasets makes it possible to examine ictal gamma dynamics in a larger and more reproducible framework. Such datasets allow simultaneous analysis at the contact, seizure, and patient levels, thereby enabling assessment not only of overall ictal gamma increase, but also of hierarchical variability and spatial informativeness (12, 13). In particular, it remains important to determine whether ictal gamma increase is merely a general feature of seizures or whether it shows preferential localization to seizure onset zone contacts (5, 14).

In the present study, we analyzed a large public SEEG dataset to quantify ictal gamma activity across contacts, seizures, and patients. We first characterized overall ictal gamma increase and its variability. We then tested whether ictal gamma increase showed preferential localization to seizure onset contacts using pooled, three-group, and within-patient analyses. To further evaluate spatial informativeness, we examined whether the top-gamma contacts within each seizure were enriched in seizure onset contacts and whether this enrichment recurred across seizures within individual patients. Finally, we examined seizure-level associations with duration and spatial concentration and quantified hierarchical variability using mixed-effects modeling.

## 2 Materials and methods

### 2.1 Dataset and study design

**Figure 1:**
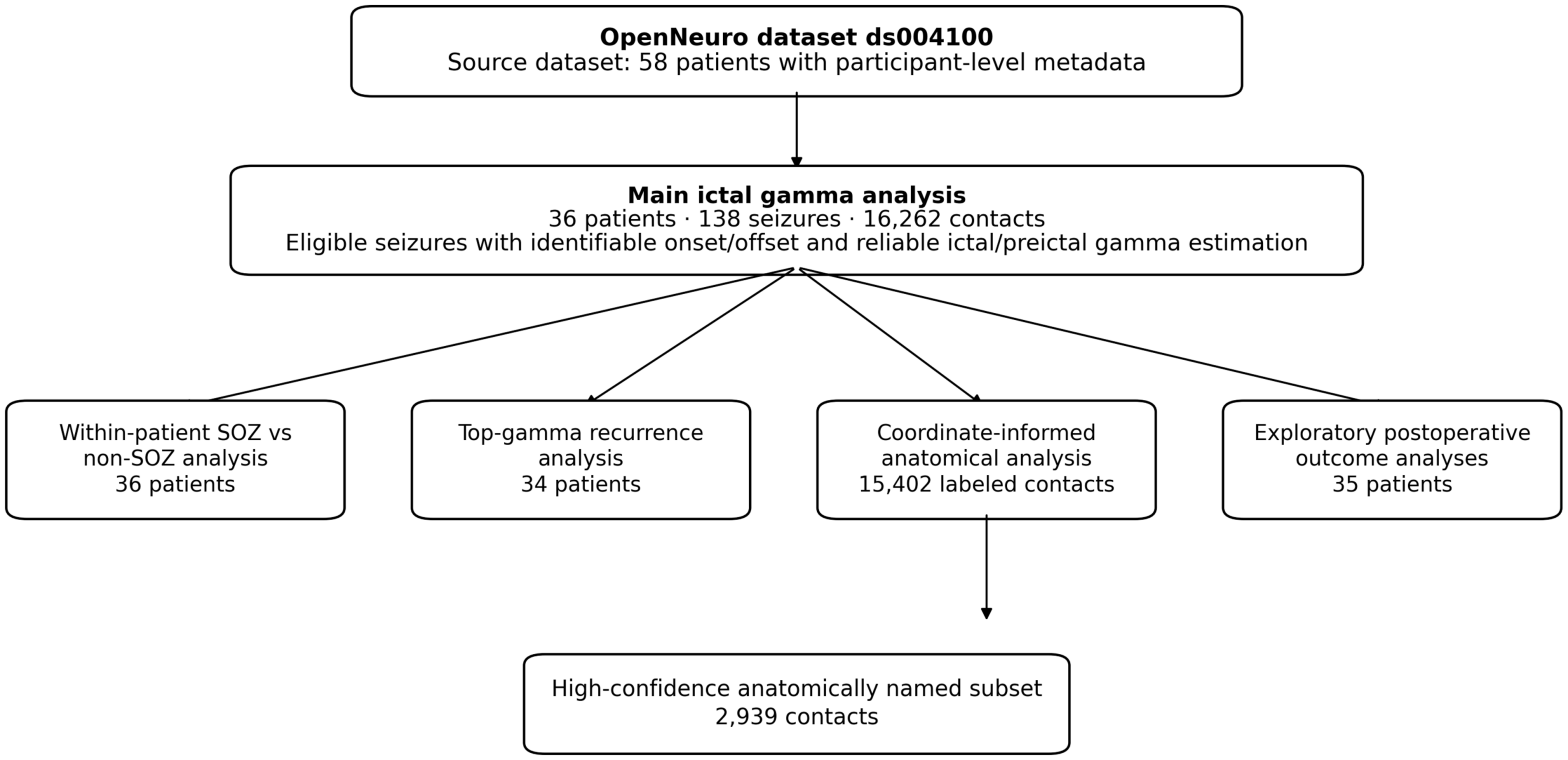
Study flow chart and analytical subsets. Intracranial EEG recordings were obtained from the public OpenNeuro dataset ds004100. The source dataset contained 58 patients with participant-level metadata in participants.tsv. After application of predefined eligibility criteria for ictal gamma quantification, the main analytical cohort comprised 36 patients, 138 seizures, and 16,262 contacts. Different downstream analyses used partially overlapping subsets according to data availability, including within-patient SOZ versus non-SOZ analysis, top-gamma recurrence analysis, coordinate-informed anatomical analysis, a high-confidence anatomically named subset, and exploratory postoperative outcome analyses.

Intracranial EEG recordings were obtained from the publicly available OpenNeuro dataset ds004100, which contains presurgical invasive recordings from patients with drug-resistant epilepsy. All data were de-identified before public release. Source dataset characteristics, including participant-level demographic and clinical metadata, were summarized from the accompanying participants.tsv file and are presented in Table 1. The overall study flow and analytical subsets are summarized in Figure 1.

**Table 1:** Source dataset characteristics and analysis subsets used in the present study.

Characteristic	Value
Source dataset characteristics (participants.tsv)	
Patients with available participant-level metadata, *n*	58
Age, years, median (IQR)	33.5 (25.0–41.8)
Age at seizure onset, years, median (IQR)	14.5 (6.0–23.8)
Female sex, *n* (%)	31 (53.4)
Male sex, *n* (%)	27 (46.6)
Left-handed/right-handed/missing, *n*	26/17/15
Non-lesional/lesional/missing, *n*	30/27/1
Ablation/resection, *n*	29/29
SEEG/ECoG, *n*	38/20
Temporal/MTL/frontal/other targets, *n*	25/17/10/6
Analysis subsets used in the present study	
Patients in main ictal gamma analysis, *n*	36
Seizures in main ictal gamma analysis, *n*	138
Contacts in main ictal gamma analysis, *n*	16,262
Patients in within-patient SOZ vs. non-SOZ analysis, *n*	36
Patients in top-gamma recurrence analysis, *n*	34
Contacts with anatomical labels, *n*	15,402
High-confidence anatomically named contacts, *n*	2,939

IQR, interquartile range; SOZ, seizure onset zone; SEEG, stereoelectroencephalography; ECoG, electrocorticography; MTL, mesial temporal lobe. Source dataset characteristics were summarized from the publicly available participants.tsv file of OpenNeuro dataset ds004100. The main ictal gamma analyses were performed in a screened analytical cohort of 36 patients, 138 seizures, and 16,262 contacts. Because different analyses required different levels of metadata availability, the effective sample size varied across downstream analysis modules; the overall study flow and analytical subsets are summarized in Figure 1.

The present study was designed to quantify ictal gamma dynamics across contact, seizure, and patient levels and to evaluate their spatial relationship with clinically defined seizure onset zone (SOZ) contacts. For the main quantitative analysis, seizures were included if they had clearly identifiable electrographic onset and offset and if the corresponding recordings allowed reliable estimation of both ictal and preictal gamma power. Seizures or contacts were excluded from quantitative analysis when required metadata were missing or when signal quality was insufficient for gamma estimation.

Because different analyses required different levels of available metadata, the effective sample size varied across analysis modules. Localization analyses were restricted to contacts with available SOZ and resection annotations. Coordinate-informed anatomical analyses were performed in the subset of contacts that could be matched to fsaverage-space electrode coordinates and assigned a coarse anatomical label.

After application of these criteria, the main analytical cohort comprised 36 patients, 138 seizures, and 16,262 contacts. Of these, 15,402 contacts received anatomical labels and were included in region-stratified analyses. A high-confidence anatomically named subset comprising 2,939 contacts was further used for sensitivity-oriented anatomical interpretation. Postoperative outcome data were available at the patient level for 35 of the 36 patients in the main analytical cohort and were used for exploratory outcome analyses. Thus, Table 1 and Figure 1 distinguish source dataset characteristics from the final analytical subsets used for seizure-level, within-patient, anatomical, and exploratory outcome analyses.

### 2.2 Gamma power quantification

Intracranial EEG recordings were obtained from the publicly available EDF files in the BIDS-formatted OpenNeuro dataset ds004100. Seizure onset and offset times were identified from the corresponding event annotation files, and channel-level metadata were obtained from the accompanying channels.tsv files. Channels labeled as bad in the channels.tsv files were excluded from analysis. Non-cerebral channels, such as EKG channels, were also excluded where applicable. Recordings were analyzed using the channel configuration provided in the public dataset, and no additional uniform re-referencing procedure was applied across all recordings. Gamma-band activity was defined as spectral power in the 30–80 Hz range. This range was selected to capture conventional gamma activity while limiting contamination from broader high-frequency activity and high-frequency oscillations, which may reflect partially distinct electrophysiological phenomena in epilepsy research. Accordingly, the present study focused on conventional ictal gamma dynamics rather than the full spectrum of high-gamma or high-frequency oscillatory activity (15). For each recording, the available sampling frequency was taken directly from the raw data, and gamma-band power was estimated using Welch-based spectral estimation within the specified analysis window. For each contact, ictal gamma power was estimated over the full electrographic seizure period, from seizure onset to seizure offset. Baseline gamma power was defined using the 5-min preictal epoch preceding seizure onset.

Gamma change was quantified as the log2-transformed ratio of ictal gamma power to baseline gamma power, expressed as log2(ictal gamma power/baseline gamma power). Positive values indicated increased gamma activity during seizures relative to baseline. Because preictal baseline activity may be influenced by interictal abnormalities, evolving preictal dynamics, medication effects, and unmeasured vigilance-state variation, baseline-normalized gamma measures should be interpreted with these potential sources of variability in mind.

### 2.3 Multi-level summaries of ictal gamma activity

At the contact level, a log2 ictal-to-baseline gamma ratio was calculated for each analyzed contact. At the seizure level, contact-level log-ratios were averaged within each seizure to obtain a seizure-wise summary of ictal gamma recruitment. At the patient level, seizure-level means were averaged within each patient to quantify inter-individual variability.

### 2.4 SOZ-based contact classification

For localization analyses, channel-level gamma features were merged with seizure onset zone and resection annotations. Contacts were classified in two ways. First, a binary classification was used, consisting of SOZ and non-SOZ contacts. Second, a three-level classification was used, consisting of non-SOZ, SOZ only, and resected SOZ contacts. These classifications were used to test whether ictal gamma increase preferentially localized to seizure onset contacts and whether resected SOZ contacts differed from non-resected SOZ contacts.

### 2.5 Within-patient SOZ versus non-SOZ analysis

To reduce the influence of between-patient differences in signal scale and electrode sampling, a within-patient paired analysis was performed in patients who had both SOZ and non-SOZ contacts. For each patient, mean and median log-ratio values were calculated separately for SOZ and non-SOZ contacts. These subject-level summaries were then compared using paired non-parametric testing.

### 2.6 Top-gamma contact enrichment and recurrence

To further evaluate the spatial informativeness of ictal gamma activity, contacts within each seizure were ranked according to gamma increase. Among contacts with positive log-ratio values, the top 10% were defined as top-gamma contacts. We then tested whether top-gamma contacts were enriched in SOZ contacts.

To evaluate recurrence across seizures within patients, we calculated how frequently each contact entered the top-gamma set across seizures from the same patient. Subject-level summaries of top-gamma entry frequency were then compared between SOZ and non-SOZ contacts.

### 2.7 Seizure duration and spatial concentration

Seizure duration was quantified for each seizure from electrographic onset to offset. To characterize spatial organization, contacts within each seizure were ranked by positive gamma increase. Spatial concentration was defined as the fraction of total positive gamma increase contributed by the top 10% of contacts in that seizure. Higher values indicated that positive gamma activity was concentrated in a smaller subset of sampled contacts.

### 2.8 Variance decomposition using mixed-effects modeling

To quantify hierarchical sources of variability in ictal gamma activity, we fitted linear mixed-effects models with random intercepts for patients and seizures. Variance components were used to estimate the relative contribution of patient-level, seizure-level, and residual variation.

### 2.9 Coordinate-informed anatomical localization and regional stratification

For anatomical analysis, channel-level gamma features and SOZ/resection annotations were merged with electrode coordinate files in fsaverage space. Contact coordinates were first matched to channel identities at the subject level. High-confidence naming-based assignments were retained for a limited set of contacts with clear anatomical meaning, including mesial temporal, insular, and cingulate contacts. For the remaining contacts, coarse cortical localization was assigned by nearest-label mapping to the fsaverage Desikan–Killiany aparc annotation. Contacts were grouped into coarse anatomical categories, including frontal, mesial temporal, lateral temporal, insular, parietal, central sensorimotor, cingulate, occipital, and unknown regions. Because localization was performed in template space and summarized at a coarse regional level, these analyses were interpreted as region-level rather than nucleus-level anatomical analyses.

### 2.10 Statistical analysis

Contact-level gamma log-ratio values were non-normally distributed. Therefore, pooled contact-level non-parametric comparisons were used primarily as descriptive summaries of group differences. Because contacts were nested within seizures and patients, the primary inferential analyses were based on hierarchical mixed-effects models that accounted for this dependence structure.

For the main SOZ versus non-SOZ comparison, contact-level gamma log-ratio was modeled as a function of SOZ status, with random intercepts for patient and seizure-within-patient. For the three-group analysis, contact group (non-SOZ, SOZ only, resected SOZ) was entered as a fixed effect in an analogous mixed-effects framework. Effect estimates with corresponding confidence intervals and *p* values from these models were used as the primary inferential results.

Binary SOZ versus non-SOZ pooled comparisons were additionally summarized using the Wilcoxon rank-sum test. Three-group pooled comparisons were additionally summarized using the Kruskal-Wallis test followed by pairwise Wilcoxon rank-sum tests. Within-patient SOZ versus non-SOZ comparisons were performed using the Wilcoxon signed-rank test. Enrichment of top-gamma contacts in SOZ was tested using Fisher’s exact test. Associations between seizure-level gamma increase and seizure duration or spatial concentration were assessed using Spearman correlation. All tests were two-sided, and *p* < 0.05 was considered statistically significant.

For anatomical stratification analyses, contact-level gamma log-ratios were compared between SOZ and non-SOZ contacts within each anatomical region using the Wilcoxon rank-sum test, with Benjamini-Hochberg false discovery rate correction across regions. Because region-specific sampling was clinically driven and uneven, these stratified analyses were interpreted as anatomically informed descriptive comparisons rather than the primary inferential framework.

## 3 Results

### 3.1 Overall increase in ictal gamma activity

A total of 16,262 contacts, 138 seizures, and 36 patients were included in the analysis. Across the dataset, gamma power during seizures was elevated relative to preictal baseline. The distribution of contact-level log-ratio values was right-skewed, indicating that although many contacts showed modest increases, a smaller subset showed marked ictal gamma elevation.

At the seizure level, the mean gamma increase was 1.72 ± 2.08 on the log2 scale, indicating substantial heterogeneity across seizures. Considerable variation was also observed across patients, suggesting marked inter-individual differences in ictal gamma dynamics.

### 3.2 Higher ictal gamma increase in SOZ than non-SOZ contacts

**Figure 2:**
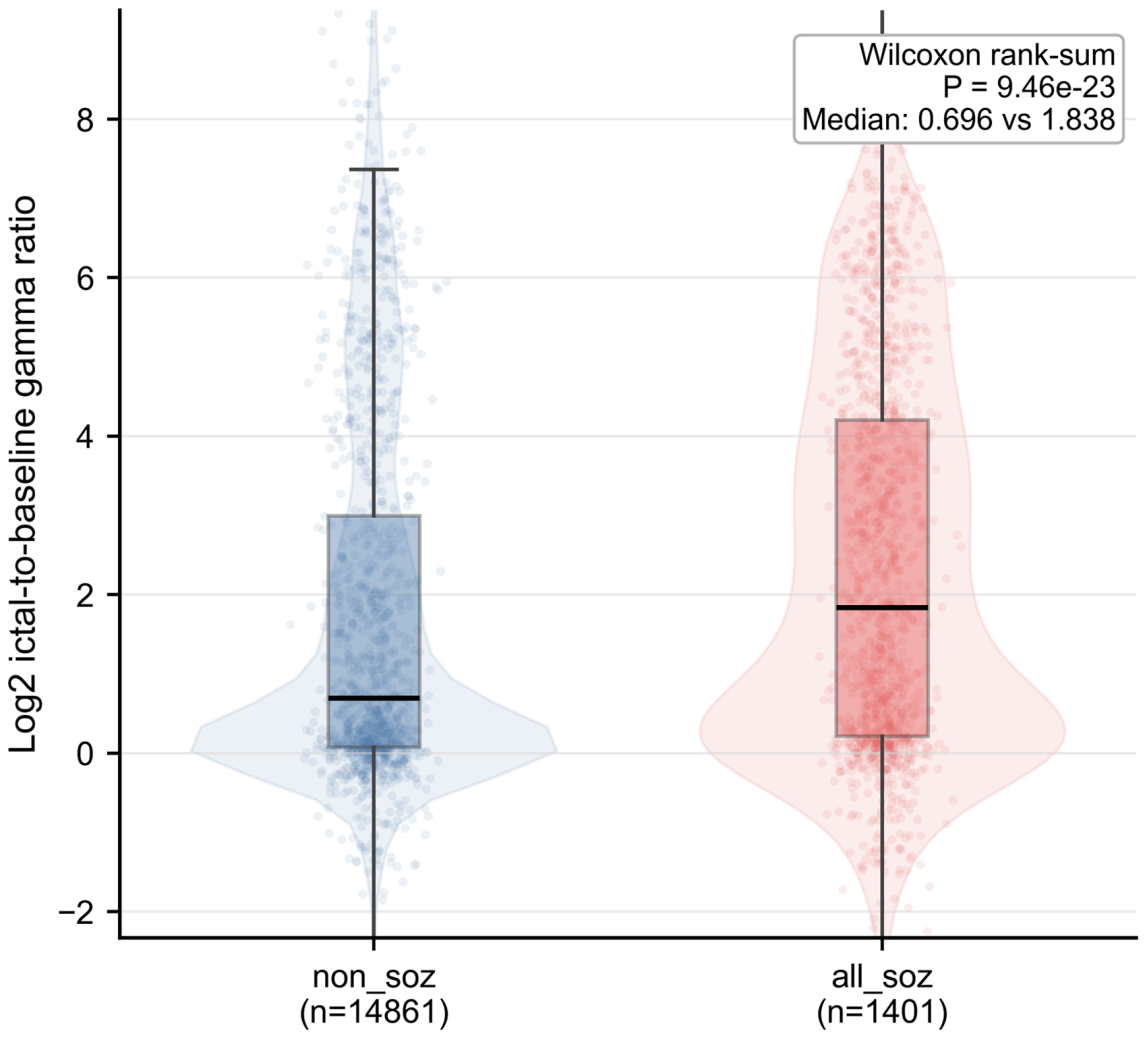
Higher ictal gamma increase in SOZ than non-SOZ contacts. Violin plots with overlaid boxplots and sampled contact-level points showing the distribution of log_2_ ictal-to-baseline gamma ratios in non-SOZ and SOZ contacts. SOZ contacts showed significantly higher ictal gamma increase than non-SOZ contacts (Wilcoxon rank-sum test, *p* = 9.46 × 10^−23^). Boxes indicate the interquartile range and median.

To descriptively summarize pooled contact-level group differences, we first compared all SOZ contacts with all non-SOZ contacts. Among the 16,262 included contacts, 14,861 were classified as non-SOZ and 1,401 as SOZ. SOZ contacts showed substantially higher log-ratio values than non-SOZ contacts (Figure 2). In the non-SOZ group, the mean log-ratio was 1.781, the median was 0.696, and the interquartile range was 0.077–2.996. In the SOZ group, the mean log-ratio was 2.342, the median was 1.838, and the interquartile range was 0.216–4.201. This difference was highly significant (Wilcoxon rank-sum test, *p* = 9.46 × 10^-23).

To account for the hierarchical dependence of contacts nested within seizures and patients, we additionally fitted a hierarchical mixed-effects model in the subset with complete data for this analysis, with random intercepts for patient and for seizure nested within patient. In this model, SOZ status remained significantly associated with higher contact-level gamma log-ratio values (*β* = 0.802, 95% CI 0.739 to 0.864, *p* < 2 × 10^-16), indicating that the main SOZ-related effect remained robust after accounting for within-seizure and within-patient dependence.

### 3.3 SOZ-related contact groups differ in ictal gamma increase

**Figure 3:**
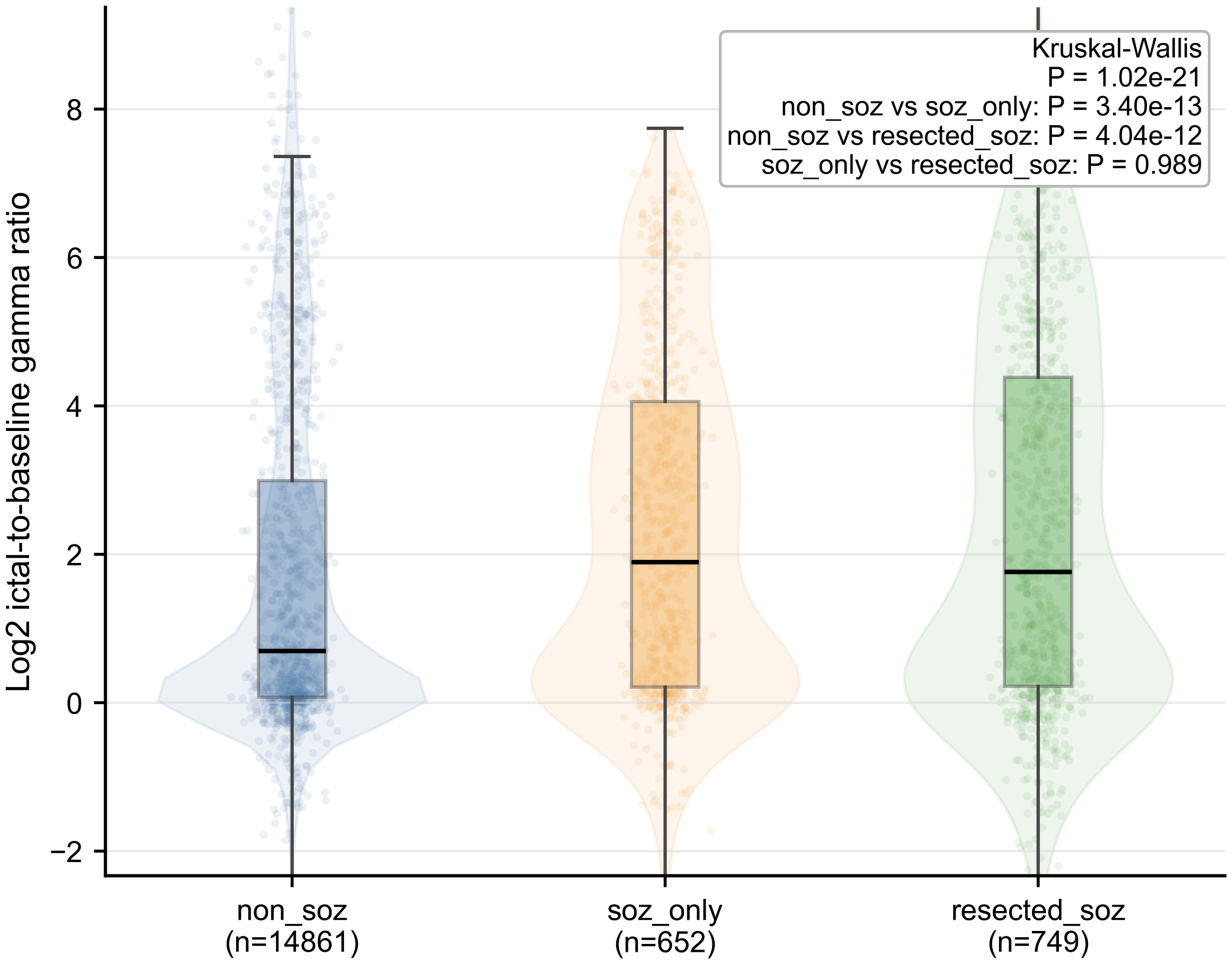
SOZ-related contact groups differ in ictal gamma increase. Violin plots with overlaid boxplots and sampled contact-level points showing log_2_ ictal-to-baseline gamma ratios across non-SOZ, SOZ only, and resected SOZ contacts. Overall group differences were significant (Kruskal-Wallis test, *p* = 1.02 × 10^−21^). Pairwise comparisons showed that both SOZ only and resected SOZ contacts differed from non-SOZ contacts, whereas SOZ only and resected SOZ contacts did not differ.

When contacts were further divided into non-SOZ, SOZ only, and resected SOZ groups, the overall difference remained significant (Kruskal-Wallis H = 96.67, *p* = 1.02 × 10^-21; Figure 3).

Descriptive statistics were as follows: non-SOZ, *n* = 14,861, mean = 1.781, median = 0.696, IQR = 0.077–2.996; SOZ only, *n* = 652, mean = 2.306, median = 1.898, IQR = 0.211–4.059; resected SOZ, *n* = 749, mean = 2.374, median = 1.764, IQR = 0.225–4.388.

Pairwise comparisons showed that both SOZ-related groups differed significantly from non-SOZ contacts. The difference between non-SOZ and SOZ only was significant (*p* = 3.40 × 10^-13), as was the difference between non-SOZ and resected SOZ (*p* = 4.04 × 10^-12). By contrast, no significant difference was observed between SOZ only and resected SOZ (*p* = 0.989).

### 3.4 Within-patient SOZ effect remains significant

**Figure 4:**
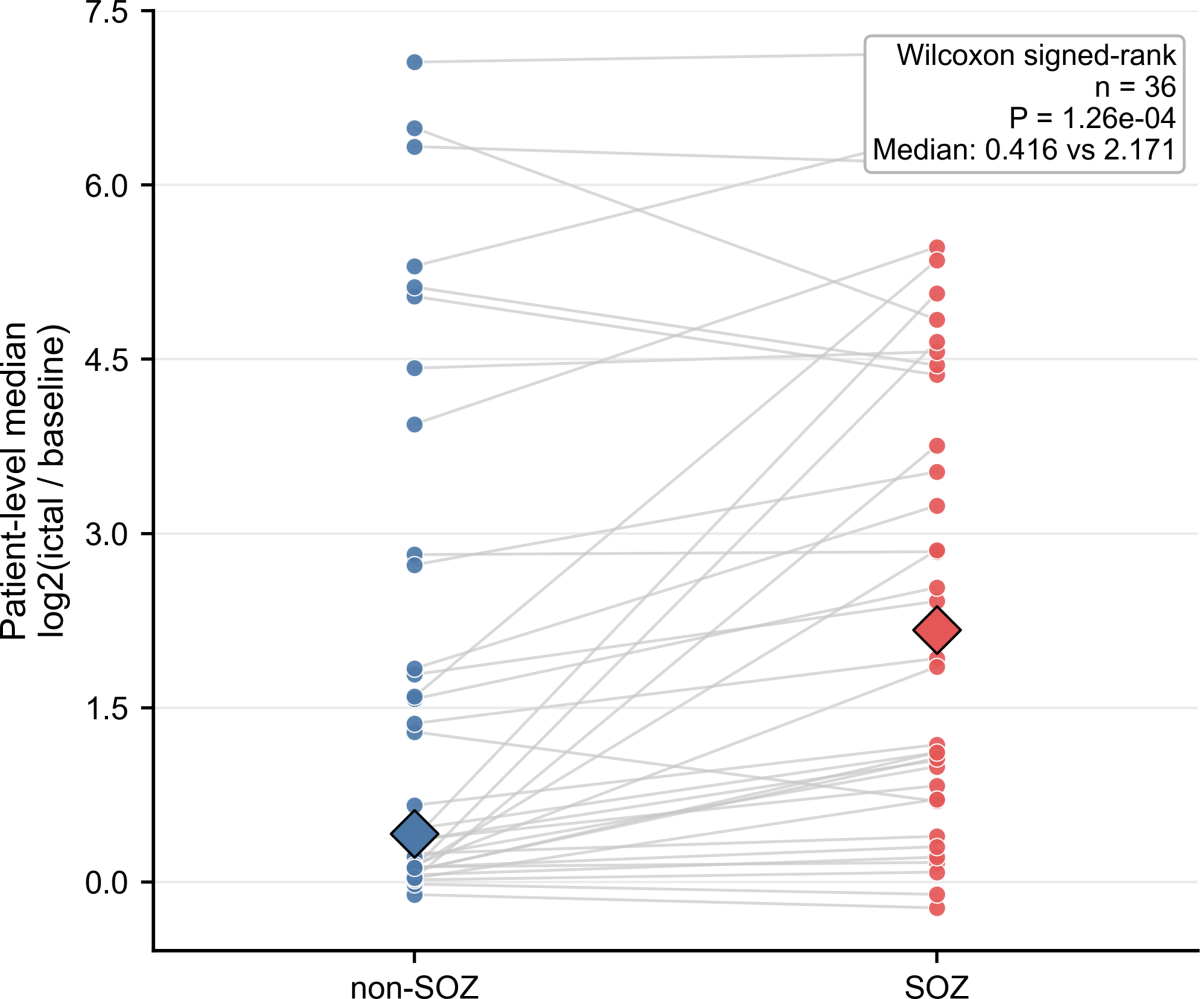
Within-patient SOZ effect remains significant. Paired plot showing patient-level median log_2_ ictal-to-baseline gamma ratios for non-SOZ and SOZ contacts in patients with both contact types available. Each line represents one patient. Diamonds indicate group medians across patients. SOZ contacts remained significantly higher than non-SOZ contacts at the within-patient level (Wilcoxon signed-rank test, *n* = 36, *p* = 1.26 × 10^−4^).

To test whether the SOZ-related effect persisted within individual patients, a paired within-patient analysis was performed in patients who had both SOZ and non-SOZ contacts. Thirty-six patients met this criterion. At the patient-summary level, the median of patient-specific SOZ medians was 2.171, compared with 0.416 for patient-specific non-SOZ medians (Figure 4). This difference was significant (Wilcoxon signed-rank test, *p* = 1.26 × 10^-4). Likewise, the median of patient-specific SOZ means was 2.553, compared with 0.852 for patient-specific non-SOZ means (*p* = 5.54 × 10^-7).

These results indicate that the SOZ effect was not merely driven by pooled contact-level analyses, but remained evident within individual patients.

### 3.5 Top-gamma contacts are strongly enriched in SOZ

**Figure 5:**
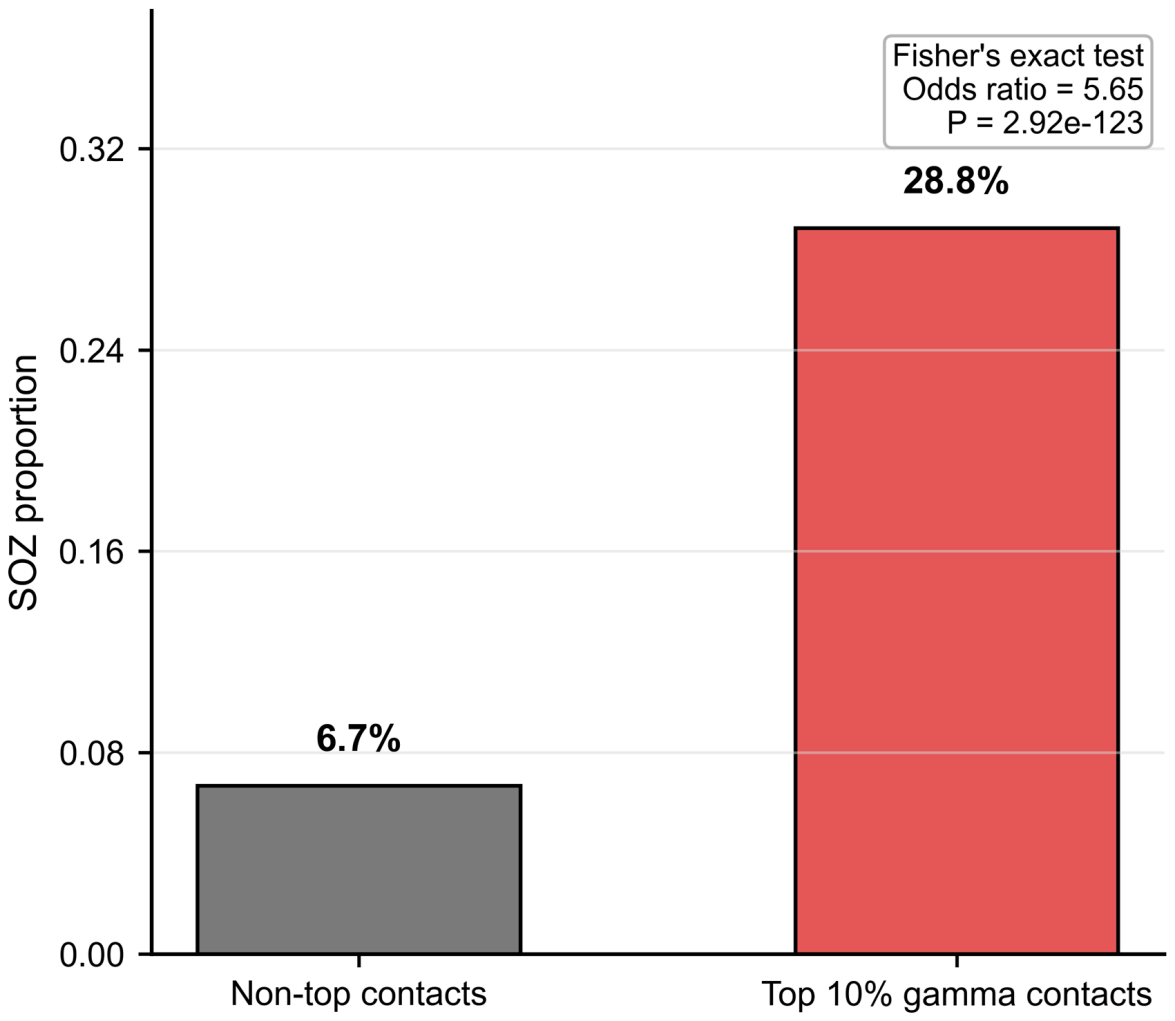
SOZ enrichment among top-gamma contacts. Bar plot showing the proportion of SOZ contacts among non-top contacts and among the top 10% gamma contacts within seizures. Top-gamma contacts were markedly enriched in SOZ contacts (28.8% vs. 6.7%; Fisher’s exact test, odds ratio = 5.65, *p* = 2.92 × 10^−123^).

To further assess the spatial informativeness of ictal gamma activity, we identified the top 10% of positive gamma contacts within each seizure and tested whether these contacts were preferentially associated with SOZ labeling. Overall, top-gamma contacts were markedly enriched in SOZ contacts (Figure 5). Among top 10% contacts, the SOZ rate was 28.8%, compared with 6.7% among non-top contacts, corresponding to an odds ratio of 5.65 (Fisher’s exact test, *p* = 2.92 × 10^-123).

**Figure 6:**
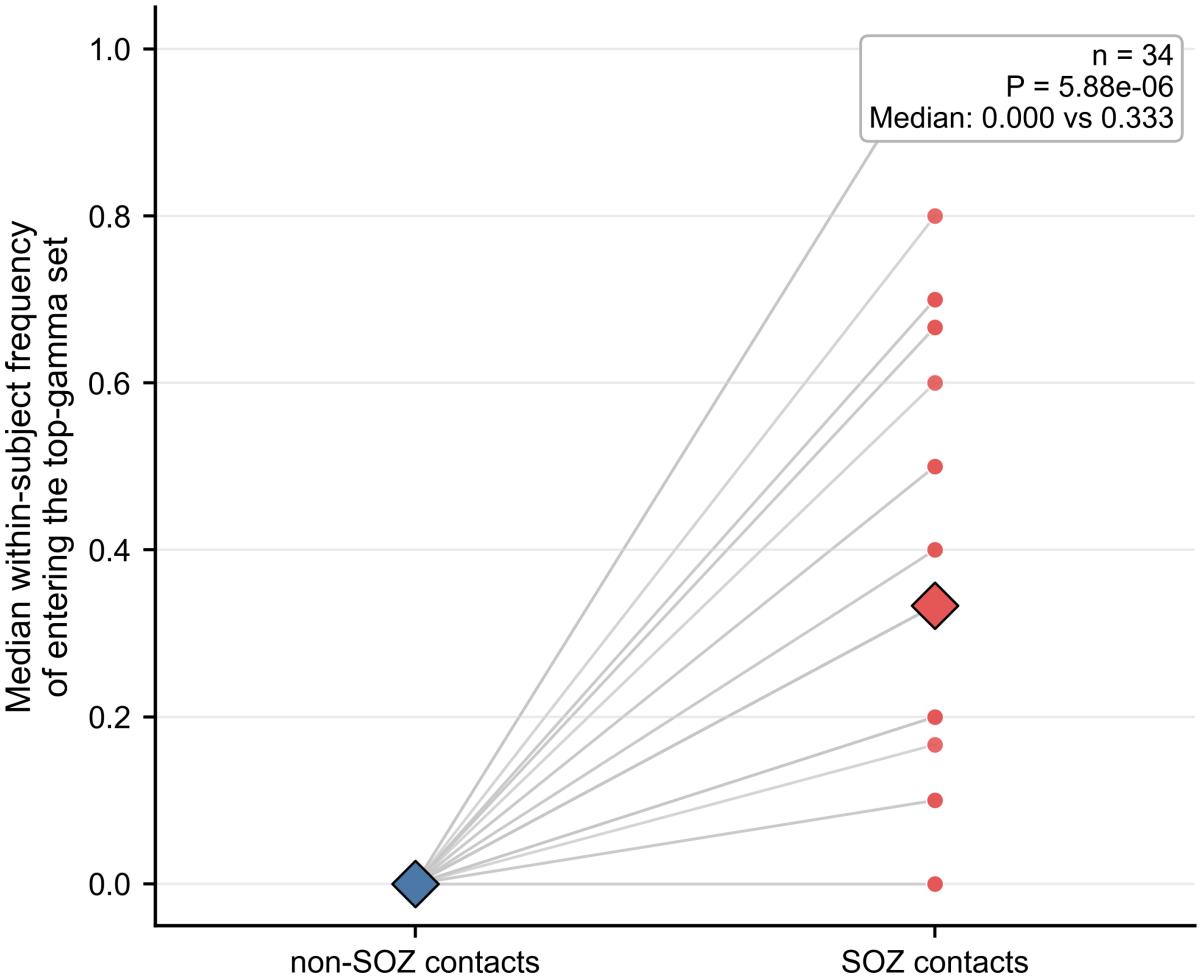
Recurrence of top-gamma contacts within patients. Paired plot showing subject-level median within-subject frequencies of entering the top-gamma set for non-SOZ and SOZ contacts. Each line represents one patient, and diamonds indicate group medians across patients. SOZ contacts entered the top-gamma set more frequently than non-SOZ contacts across seizures within patients (Wilcoxon signed-rank test, *n* = 34, *p* = 5.88 × 10^−6^).

In 34 patients with sufficient data for subject-level comparison, the median of subject-specific median top-gamma frequencies was 0.333 for SOZ contacts and 0 for non-SOZ contacts (Figure 6). This difference was significant (Wilcoxon signed-rank test, *p* = 5.88 × 10^-6), indicating that SOZ contacts were more likely than non-SOZ contacts to repeatedly appear among the highest-gamma contacts across seizures.

Taken together, these results suggest that ictal gamma activity is not only stronger in SOZ contacts on average, but that the most prominent gamma contacts are preferentially concentrated in SOZ and tend to recur there across seizures within patients, although the degree of recurrence remains heterogeneous across individuals.

### 3.6 Relationship with seizure duration and spatial concentration

Seizure duration was not significantly associated with seizure-level mean gamma increase (Spearman *ρ* = 0.037, *p* = 0.668), indicating that stronger ictal gamma recruitment was not simply a consequence of longer seizures.

By contrast, mean seizure-level gamma increase was inversely associated with spatial concentration (Spearman ρ = −0.32, *p* = 1.3 × 10^-4), indicating that seizures with stronger gamma activity tended to show a broader spatial distribution of positive gamma increase rather than a more focal pattern.

### 3.7 Patient-level differences dominate variability in ictal gamma activity

Variance decomposition using linear mixed-effects modeling showed that patient-level differences accounted for 67.02% of total variance in ictal gamma activity, whereas seizure-level differences accounted for 10.46%, and the remaining 22.52% was attributed to residual variability. These findings indicate that inter-individual variability was the dominant source of heterogeneity in ictal gamma dynamics.

### 3.8 Coordinate-informed anatomical stratification of ictal gamma activity

Coordinate-informed anatomical localization was achieved for most analyzed contacts by integrating fsaverage-space electrode coordinates with coarse regional anatomical mapping. Among 16,262 contacts from 36 patients, 15,402 contacts received anatomical labels. The largest sampled regions were frontal (*n* = 3,742), mesial temporal (*n* = 3,222), lateral temporal (*n* = 2,614), insular (*n* = 1,642), parietal (*n* = 1,095), and central sensorimotor (n = 963) contacts, consistent with clinically uneven electrode sampling across anatomical regions.

Region-stratified comparisons showed that SOZ contacts had significantly higher gamma log-ratio values than non-SOZ contacts in several anatomical regions after false discovery rate correction. The strongest separation was observed in mesial temporal contacts, where the median gamma log-ratio was 2.15 in SOZ contacts and 0.58 in non-SOZ contacts (FDR-corrected *p* = 7.38 × 10^-21). Significant SOZ-related increases were also observed in parietal contacts (median 3.13 vs. 1.10, FDR-corrected *p* = 5.33 × 10^-10), central sensorimotor contacts (median 3.05 vs. 1.80, FDR-corrected *p* = 2.25 × 10^-6), insular contacts (median 2.65 vs. 0.79, FDR-corrected *p* = 3.24 × 10^-5), and lateral temporal contacts (median 1.44 vs. 0.56, FDR-corrected *p* = 1.30 × 10^-3). By contrast, frontal contacts did not show a significant SOZ versus non-SOZ difference (median 0.30 vs. 0.32, FDR-corrected *p* = 0.99). Cingulate contacts showed a non-significant trend, and occipital contacts were not formally compared because no SOZ contacts were present in that region.

These findings indicate that SOZ-related differences in ictal gamma activity were not spatially uniform across sampled regions, with the most robust separation observed in mesial temporal and selected extra-temporal regions. A detailed summary of region-stratified SOZ versus non-SOZ comparisons is provided in Table 2. These region-specific patterns should be interpreted cautiously because electrode coverage was clinically driven and uneven across regions, and because the anatomical analyses were based on coarse template-space regional assignments rather than precise subject-specific localization.

**Table 2:** Coordinate-informed region-stratified comparison of ictal gamma log-ratio between SOZ and non-SOZ contacts.

Region	Region group	n SOZ	n non-SOZ	Median SOZ	Median non-SOZ	Mean SOZ	Mean non-SOZ	*P* value	FDR-corrected P
Mesial temporal	Temporal	482	2,740	2.148	0.579	2.559	1.524	9.22 × 10^-22	7.38 × 10^-21
Unknown	Unknown	299	1,135	2.161	0.402	2.423	1.327	3.18 × 10^-12	1.27 × 10^-11
Parietal	Parietal	64	1,031	3.133	1.104	3.684	1.863	2.00 × 10^-10	5.33 × 10^-10
Central sensorimotor	Central	46	917	3.054	1.802	3.765	2.210	1.12 × 10^-6	2.25 × 10^-6
Insula	Insula	117	1,525	2.647	0.796	2.610	1.882	2.03 × 10^-5	3.24 × 10^-5
Lateral temporal	Temporal	69	2,545	1.437	0.556	1.787	1.580	9.74 × 10^-4	0.001
Cingulate	Cingulate	23	423	0.432	0.143	1.012	1.316	0.071	0.082
Frontal	Frontal	286	3,456	0.296	0.325	1.399	1.410	0.991	0.991
Occipital	Occipital	0	244	—	0.448	—	0.874	—	—

SOZ, seizure onset zone; FDR, false discovery rate. Gamma values are presented as median log2 ictal-to-baseline gamma ratios within each anatomical region. P values were obtained using Wilcoxon rank-sum tests comparing SOZ and non-SOZ contacts within each region. FDR-corrected p values were calculated using the Benjamini–Hochberg procedure. Occipital contacts were not formally compared because no SOZ contacts were present in that region. Unknown indicates contacts that could not be confidently assigned to a coarse anatomical region after coordinate-informed mapping.

### 3.9 Exploratory postoperative outcome analyses

Postoperative outcome data were available for 35 of 36 patients in the main analytical cohort, including 20 patients with favorable outcome and 15 with unfavorable outcome. Exploratory patient-level analyses did not identify significant associations between postoperative outcome and overall ictal gamma magnitude, SOZ-versus-non-SOZ gamma difference, or top-gamma recurrence measures. Likewise, within outcome strata, ictal gamma values did not significantly differ between resected and unresected SOZ contacts. These exploratory findings do not support a clear prognostic association of the current gamma metrics at the patient level and should be interpreted as hypothesis-generating rather than clinically definitive.

Several factors may explain this negative result. The present gamma metrics may reflect seizure onset-related activation more directly than resection adequacy, whereas postoperative outcome depends on broader network extent, surgical strategy, and whether all clinically relevant seizure-generating tissue was removed. In addition, the current outcome analyses were exploratory and limited by patient-level outcome coding and the absence of subject-specific native-space resection cavity mapping.

## 4 Discussion

The main finding of this study is that ictal gamma increase was not only robustly elevated during seizures, but was also spatially informative, being consistently greater in SOZ than in non-SOZ contacts. This effect remained evident in three-group analyses, within-patient paired comparisons, and enrichment analyses of top-gamma contacts. Thus, the present results suggest that ictal gamma increase is more than a generic ictal phenomenon and is consistently associated with seizure onset-related contacts in a large SEEG dataset (16, 17). This interpretation is broadly consistent with prior intracranial studies suggesting that seizure-related gamma activity tends to be enhanced in seizure-onset or early propagation territories (16, 17). At the same time, the present study extends this literature by showing, in a larger public SEEG cohort, that the strength of this association is markedly heterogeneous across patients and is not spatially uniform across sampled anatomical regions. Potential reasons for these differences across studies include variation in electrode coverage, differences in analytical scale, and differences in the frequency range used to define seizure-related high-frequency activity.

The SOZ-related findings are important for several reasons. First, the pooled contact-level comparison showed a clear separation between SOZ and non-SOZ contacts, with substantially higher median gamma increase in SOZ. Second, this difference remained significant within patients, reducing the likelihood that the result was driven solely by between-patient differences in implantation strategy or signal scale. Third, the top-gamma enrichment analysis extended the localization result beyond a simple group mean effect. The highest-gamma contacts within seizures were strongly enriched in SOZ, and SOZ contacts entered the top-gamma set more frequently across seizures within patients. Together, these results suggest that ictal gamma increase is consistently associated with a spatially recurrent pattern linked to seizure onset-related contacts.

The lack of a detectable difference between SOZ only and resected SOZ contacts is also informative. Within the current dataset, the magnitude of ictal gamma increase appeared to be linked primarily to SOZ status itself rather than to resection status among SOZ contacts. This may indicate that gamma-based differences are associated with SOZ status itself rather than with resection status among SOZ contacts, but do not, by themselves, distinguish whether a given SOZ contact was ultimately included in the resection field (18, 19).

Another important finding was the marked heterogeneity in ictal gamma dynamics across patients (20). Mixed-effects variance decomposition showed that patient-level differences accounted for most of the total variance, far exceeding seizure-level variability. This suggests that although ictal gamma increase is a robust group-level phenomenon, its absolute magnitude is strongly shaped by inter-individual factors (21, 22). In practice, this implies that gamma-based biomarkers may be most informative when interpreted in a patient-specific context rather than through a single universal threshold (23).

The seizure-level analyses provided additional context for interpreting these spatial results. Seizure duration was not associated with mean ictal gamma increase, suggesting that stronger gamma recruitment was not simply a consequence of longer seizures. By contrast, the inverse relationship between seizure-level gamma increase and spatial concentration indicates that seizures with stronger gamma activity tended to recruit a broader set of sampled contacts. This pattern argues against a simplistic interpretation in which stronger ictal gamma merely reflects a tiny focal hotspot. Instead, stronger gamma increase may accompany more extensive ictal recruitment while still remaining preferentially expressed in onset-related contacts (24, 25).

The anatomical stratification analyses provide additional spatial context for interpreting these findings. Rather than showing a uniform SOZ-related effect across all sampled regions, ictal gamma increase demonstrated marked regional heterogeneity (4). The strongest SOZ versus non-SOZ separation was observed in mesial temporal contacts, with additional significant effects in insular, parietal, central sensorimotor, and lateral temporal regions, whereas frontal contacts showed no detectable SOZ-specific increase despite dense sampling. This pattern suggests that SOZ-related gamma differences may vary across coarse anatomical regions, but these findings should be interpreted as region-level associations derived from template-space mapping rather than precise anatomical localization (26).

Several limitations should be acknowledged. First, although coordinate-informed anatomical localization was incorporated in the present study, localization was performed at a coarse regional level in template space rather than by subject-specific native-space anatomical segmentation. Accordingly, these analyses support regional anatomical interpretation but do not provide precise nucleus-level or patient-specific structural localization. In addition, the present analysis focused on the conventional 30–80 Hz gamma band and therefore does not capture the broader spectrum of high-gamma or high-frequency oscillatory activity, which may carry partially distinct seizure-related information. A broader 30–200 Hz range might capture additional high-gamma and other high-frequency seizure-related components, potentially altering both the magnitude and spatial distribution of the observed effects; however, such an approach would also broaden the signal definition beyond conventional gamma activity. The 5-min preictal baseline may still be influenced by interictal abnormalities, evolving preictal dynamics, medication effects, and unmeasured vigilance-state variation, and the present dataset did not permit reliable uniform control of sleep/wake state across all analyzed seizures. Second, postoperative outcome data were available at the patient level for most patients in the analytical cohort, but exploratory analyses did not identify a clear association between the current gamma metrics and postoperative outcome. In addition, subject-specific native-space resection cavity mapping was not available, limiting direct evaluation of the spatial overlap between gamma-defined localization and the actual surgical resection volume. Third, electrode implantation in SEEG is clinically driven and therefore spatially uneven across patients and brain regions. Fourth, pooled contact-level comparisons do not fully eliminate dependence among observations because contacts are nested within seizures and patients; to address this, we supplemented pooled analyses with within-patient comparisons and mixed-effects variance decomposition.

From a clinical perspective, these findings suggest that quantitative ictal gamma analysis could potentially serve as a complementary tool during intracranial EEG review by highlighting contacts with relatively strong seizure-related gamma increase and by summarizing within-seizure spatial ranking. However, such an approach should be viewed as supportive rather than replacement-level, and prospective validation would be required before adaptation to routine EMU or presurgical localization workflows.

Future work should extend these findings in at least three directions. First, subject-specific native-space electrode localization will be needed to assess anatomical specificity more rigorously and to refine these coarse template-space regional findings. Second, integration of postoperative outcome data with subject-specific resection cavity mapping will be necessary to determine whether more spatially specific gamma-derived measures have prognostic value beyond consistency with SEEG-defined SOZ labeling. Third, temporally resolved analyses may help clarify how ictal gamma recruitment evolves across seizure phases and whether gamma-derived features can support future predictive or localization models.

In summary, the present study provides a multi-level characterization of ictal gamma dynamics in a large SEEG dataset and supports the preferential localization of ictal gamma increase to seizure onset contacts.

## 5 Conclusion

Ictal gamma activity increases robustly during seizures in a large SEEG dataset, but its magnitude varies substantially across patients. Most importantly, SOZ contacts show greater ictal gamma increase than non-SOZ contacts, and the highest-gamma contacts within seizures are strongly enriched in SOZ. This SOZ-related effect is anatomically heterogeneous, with the most robust separation observed in mesial temporal and selected extra-temporal regions. These findings suggest that ictal gamma increase is a spatially informative electrophysiological feature associated with seizure onset-related contacts in intracranial recordings.

## Data Availability

Publicly available datasets were analyzed in this study. This data can be found at: Repository: OpenNeuro Accession number: ds004100 direct link: https://openneuro.org/datasets/ds004100/versions/1.1.3.
